# General Practitioners and the Impact of Low Income in the Lives of People With Epilepsy: Results of an Australian Survey

**DOI:** 10.1111/jep.70470

**Published:** 2026-05-15

**Authors:** Christine Walker, Chris L. Peterson

**Affiliations:** ^1^ Epilepsy Foundation Surrey Hills Canterbury Victoria Australia; ^2^ Epilepsy Australia Surrey Hills Canterbury Victoria Australia; ^3^ Adjunct, Department of Social Inquiry School of Social Science and Humanities La Trobe University Bundoora Victoria Australia

**Keywords:** Australia, disadvantage, effect on life, epilepsy, general practitioners, income differences, social gradient

## Abstract

**Rationale:**

This paper reports results of research undertaken with an Australian sample of people with epilepsy (PWE) of the impact of low income on their Quality of Life (QoL) level of Social Support and important life aspects.

**Aims and Objectives:**

Australian GPs have up to 10 PWE on their books. It is important that they have a good understanding of low‐income effects on health and wellbeing of patients with epilepsy.

**Methods:**

Wave 6 of the Australian Epilepsy Longitudinal Study (2022/23) is a cross‐sectional study investigating a cohort of Australian PWE aged 18 years and older. A mixed methods analysis was undertaken.

**Results:**

QoL, Social Support and a range of life factors such as relationships, self‐esteem and health were significantly worse for PWE with low incomes The qualitative component showed themes related to financial stress which emerged prior to the end of the COVID‐19 pandemic.

**Conclusion:**

GPs as first point of care providers are best placed to assist their patients’ manage their health and wellbeing. Having a greater understanding of the effects of socioeconomic status of PWE can improve GP management including making informed referrals to other health providers.

## 1 Introduction

Epilepsy is a chronic neurological disease of the brain. It affects 50 million people of all ages globally, more so in poorer countries [1]. Wigglesworth et al. [2] report that overall, in the United Kingdom, just over nine people per 1000 have epilepsy. This means that an estimated 633,000 people are living with epilepsy in the UK. In 2017–18, 0.6 percent of Australians, or 151,000 people, had epilepsy, with the same prevalence for males and females. These rates came from self‐reported diagnosis of epilepsy [3]. Epilepsy was most prevalent amongst the 65+ age group with 0.9% of people in this group These estimates being self‐reported data may underestimate epilepsy prevalence

In 2022, the International League Against Epilepsy published its results on the need for epilepsy to be strengthened in medical curricula [4]. Based on the WHO definitions of Low,‐ Middle‐ and High‐ Income countries, the authors were concerned that people with epilepsy (PWE) in low‐income countries have fewer specialists and could not afford that care. In turn, low‐income countries had the greatest numbers of PWE. In this case, educating general practitioners about the role of income in epilepsy could assist.

At the same time, it should be recognised that high‐income countries, such as the United Kingdom, United States and Australia have income inequalities. In Australia in 2016, those people in high income groups had five times more income than those in the lowest group who were most likely to be reliant on social security [5]. While the number of PWE on low incomes in high‐income countries may be lower than those in low‐income countries, they will face similar access issues.

Marmot and Allan pointed out that the coronavirus pandemic highlighted the inequalities already evident in societies and their health systems so that those in the lower social gradients with low or no incomes, facing uncertainties of paying bills or losing housing, were at greater risk of developing COVID‐19 [6, 7]. They experienced greater harm, such as hospitalisation, poor care and death. In 2014 Marmot and Allen identified the social determinants of health including housing, income inequities were as important to health as access to health services [8]. In this article the authors use the insights of Marmot and others to explore how Australian people with epilepsy coped with the social and economic constraints introduced to contain COVID‐19 and whether any income inequalities among PWE reflect a social gradient within the epilepsy population. However, Marmot emphasised that unequal access to a health system was only part of the problem.

In Australia the burden of epilepsy is unequally distributed with regard to remoteness, educational levels, income, housing and employment. Those at the greatest disadvantage are more likely to have higher risk factors [9]. For example, it may not generally be appreciated that PWE can have poor health or a premature death from epilepsy [3]. Epilepsy was the 30th greatest disease burden in Australia, and its expenditure was higher for males.

Singh et al [4] demonstrate that General Practitioners (GPs) play an important role in diagnosis and treatment of PWE. According to Starfield et al [10] GPs have a key role in the life and health of patients and this is sometimes estimated to a lesser degree than it should be. Workload of GPs has been reported at 10 PWE as the estimate on a GP's books [11]. Given that number, the effects on health and wellbeing of PWE, and in particular knowledge of the effects of low income can provide GPs with important information for decision making and treatment. The role internationally that GPs have in dealing with social and economic inequality has been discussed [12]. In Australia, people are relatively free to choose their GP, though the amount of choice declines for those in more remote areas of Australia. Some GPs bulkbill, that is, they submit the bill directly to the government funded Medicare service. While this reduces low‐income people's out‐of‐pocket costs, it can mean shorter consultations.

Cotterill et al. [13] discuss how accounting for UK patients' experiences of accessible and appropriate care, there needs to be improved integration and communication between generalists and specialists in epilepsy care. Patients in the study reported specialists may feel burdened and GPs need better knowledge of epilepsy, (particularly of patients’ psychosocial needs) in developing epilepsy services. Minshall and Neligan [14] report that in the UK care home services, having a GP with a special interest in epilepsy can lead to providing better clinical needs, reduce the need for referral and help educate carers and colleagues. GPs have a central role in epilepsy management and should be fully involved amongst other things in initiatives on training for seizure management [15].

Despite the lower numbers of PWE seen by a GP, it is important as primary providers that GPs are aware of the drawbacks to health and disadvantages that PWE face. These include challenges in many aspects of life, including with relationships, health and self‐esteem, and their need for longer consultations with GPs. Begley et al. [16] researched differences between patient experiences in one high socioeconomic (SES) clinic and three low SES clinics in the US. They found low SES PWE used emergency hospital services more and visited GPs more frequently. It was a similar case with neurologists. The low SES patients were more likely to have uncontrolled seizures, side effects of ASMs (antiseizure medicines) and poorer QoL. GPs need to understand the effects of income‐based differences in healthcare for their patients with epilepsy and how they affect their need for treatment.

In this article we use data collected in 2022/23 (Wave 6) of the Australian Epilepsy Longitudinal Study to explore these findings as they relate to people with epilepsy. The main hypothesis is that income has a decisive impact on Quality of Life (QoL), health and other key life factors for PWE [17] and GPs can play a key role by accounting for that. While GPs may diagnose epilepsy at its onset, there is little known about their role in continuity of care and how they might assist PWE on lower incomes manage their care.

## 2 Methods

The Australian Epilepsy Longitudinal Survey (AELS) uses the Australian Epilepsy Research Register (AERR), a national research register from the Epilepsy Foundation in Australia. AELS participants 18 years and older completed a paper‐based or online survey. Participation is voluntary. Surveys known as ‘Waves’ have been conducted approximately every 3 years, since 2006. Each survey collects data on income, employment, housing, marital status, education and marital status. Additionally, information on access to health care, including access to GPs, specialist care and allied health are all collected. Experience of seizures, medications, hospitalisations, psychological health and surgery are also collected. These data mean that comparisons, showing either changes or stability in people's circumstances and health status across time may be made. Additionally, each survey may offer a small section exploring a current event to see if it impacted on those living with epilepsy.

The survey mainly being reported on is Wave 6 (2022/23). Deakin University Human Ethics provided ethics approval (2013 – 011). All participants gave informed consent. Wave 5 (2019/21) is referred to in the qualitative results in the paper for comparative purposes. It was conducted between 2019/20 and 2022/23, and had 265 PWE aged 18 and older.

Wave 6 included participants’ experiences of COVID‐19. QOLIE‐31measured QoL. QOLIE‐31 and its subscales demonstrate good internal consistency and test–retest reliability. Cronbach's alpha ranged from 0.77 to 0.85 [18]. The eight‐item modified Medical Outcomes Study Social Support Survey (MOS 8) measured social support. It has very good psychometric properties and a Cronbach's alpha of 0.93 [19].

Quantitative analyses were undertaken with the statistical package SPSS Version 27 (IBM Corp. 2020, NY). Frequencies, crosstabs (using Cramer's V and Gamma), t‐tests and One‐way ANOVA were used to examine group differences in the effects of epilepsy on the lives of PWE. Effect sizes (Cohen's d and Eta squared) are presented.

Analysis of free text is now commonly used across longitudinal surveys [20, 21]. These comments are valuable as they embellish the statistical results and provide additional evidence of coping mechanisms. There was one open‐ended question: the extent of financial problems experienced during COVID‐19. In this article the influence of income level on these financial problems is presented. A thematic approach was chosen to analyse these responses. This involved line‐by‐line coding of responses independently by both authors to identify emerging themes followed by discussion of their meaning to arrive at agreed themes on managing epilepsy [22, 23]. The themes illuminate the experiences of PWE managing financial problems and changed circumstances while highlighting the levels of economic uncertainty PWE live with all the time.

## 3 Results

The response rate was 20 percent. There were 174 participants in total in Wave 6. Numbers and percentages are reported in Tables 1 and 2. Table 1 shows the average age as 50.97 years with a range 19‐90 years. There were almost two‐thirds women (65.7%), while 42.8 percent of PWE had a university qualification; 43.3 percent were employed; 25.3 percent lived alone; and 28.7 percent were renting.

**Table 1:** Sociodemographics Wave 6.

Item	Level	*n* (%)
Age	Mean: 50.97 (SD: 16.91) Range: 19–90	
	Median: 49.50	
		*Missing 0*
Gender	Female	113 (65.7%)
	Male	57 (33.1%
	Other	1 (0.6%)
	Prefer not to say	1 (0.6%)
		*Missing 0*
Education	Up to year 10	18 (10.8%)
	Year 11 and 12	34 (20.5%)
	TAFE/Trade	25 (15.1%)
	Diploma	18 (10.8%)
	University qualification	71(42.8%)
		*Missing 6*
Employed	Yes	74 (43.3%)
		*Missing 1*
	Retired	40 (23.3%)
	Studying	14 (8.1%)
	Not working due to epilepsy	38 (22.1%)
	Not working due to a disability, not epilepsy	26 (15.1%)
	Seeking paid employment	9 (5.1%)
Where living	City	104 (61.5%)
		*Missing 3*
Living arrangements	Living alone	43 (25.3%)
≥18 years	With partner and/or children	86 (50.6%)
	With other family	28 (16.5%)
	With friends/shared accommodation	4 (2.4%)
	Residential	4 (2.4%)
	Other	5 (2.9%)
		*Missing 2*
Accommodation	Fully owned home	70 (44.6%)
	Purchasing a house (mortgage)	42 (26.8%)
	Renting	45 (28.7%)
		*Missing 15*
Weekly income	$0–$250	11 (6.9%)
	$250–$499	40 (25.0%)
	$500–$749	38 (23.8%)
	$750–$999	16 (10.0%)
	$1000–$1249	20 (12.5%)
	$1250–$1499	15 (9.4%)
	$1500–$1749	3 (1.9%)
	$1750 or more	17 (10.6%)
		*Missing 12*
Centrelink recipient		79 (46.2%)
	Disability pension	53 (30%)
	Family allowance	9 (5.2%)
	Newstart	4 (2.3%)
	Age pension	8 (4.7%)
	Other	5 (2.9%)

### 3.1 Effects of Weekly Income Differences

#### 3.1.1 Income Classified as Weekly Income before Tax

Low $0–$749; Moderate $750–$1499; Well‐off $1500 or more.

There were significant differences in income, with those with a university education mostly having the highest income (Gamma 0.460 *p* < 0.001). Those on a high income were mostly seizure free (Gamma −0.349 *p* < 0.001). In addition, those with higher incomes had fewer ASMs (Gamma −0.394 *p* < 0.001).

During COVID‐19 28 (18.2%) had financial problems. This was ability to pay bills on time and ability to seek finance when owing $4000. Those with a low income had significantly more problems in accessing healthcare during COVID‐19 (Gamma 0.507 *p* < 0.001), but had significantly more government support (Gamma 0.461 *p*: 0.002). The well‐off had about 25 percent more QoL (*F*: 10.51 *p* < 0.001 eta squared 0.151). The low‐income group had significantly less Social Support (measured by MOS‐8), about 10 percent less than moderate income PWE and 20 percent less than those well‐off (*F*: 4.83 *p*: 0.010 eta squared 0.065).

#### 3.1.2 Income Differences of the Effect of Epilepsy on Dimensions of Life

The extent to which epilepsy affects 15 dimensions of life (such as relationship with partner and friends, work, health and self‐esteem) is substantially influenced by weekly income, shown in Table 2 (below). An additive scale of 15 dimensions of life affected by epilepsy had been constructed. It has a Cronbach's alpha of 0.96 and inter‐item correlation of 0.60. The less well‐off PWE are, the more epilepsy has a negative effect on their lives.

**Table 2:** Effect of income on a composite scale of life aspects.

Weekly income	*n* (%)	Mean (SD)	*F*	*p*	*η* ^2^
Low income $0–$749	20 (40.0%)	58.89 (33.80)			
Moderate $750–$1499	17 (34%)	39.09 (23.68)			
Well‐off $1500 or more	13 (26.0%)	18.46 (15.88)			
Total	50	41.64 (30.89)	9.07	< 0.001	0.279

*Note:* A high mean score refers to epilepsy having a more negative effect.

*η* ^2^ eta squared.

Scale scored 0–100.

Overwhelmingly those worse off in terms of income have the greatest negative outcomes on this composite measure. It shows that PWE with low income had more than three times the negative effect of epilepsy compared to those who were well‐off, and a third more than those with a moderate income. Differences were significant.

##### 3.1.2.1 Welfare

Welfare payments were received by 46.2 percent of PWE. There was significantly less QoL for these PWE compared to those who did not receive benefits (*t*: −3.97 df: 120 *p* < 0.001 Cohen's *d*: 0.64), and more negative impact of epilepsy on the composite range of aspects of life (*t*: 3.29 df: 50 *p*: < 0.001 Cohen's *d*: 0.94). During COVID‐19 28 (40.6%) of those on welfare payments, known in Australia as Centrelink payments compared to 16 (19.0%) of other PWE received significantly more financial support benefits (Cramer's *V*: 0.237 *p*: 0.003).

### 3.2 Open Ended Responses to “During COVID‐19, Did You Have Any Financial Problems?”

There were 25 responses, 3 of them being unclear as to their meaning and not included. In summary the themes that emerged were:

- overall levels of increased stress
- recalculation of income to meet immediate needs‐food, household utility bills
- ‘going without’ including medicines, medical appointments,
- relying on family support, Government funds, use of savings
- expressions of distress, uncertainty

Table 3 shows those who provided details of their experiences of lockdowns, were all on low or moderate incomes, some on government pensions, while others were in casual work. Some casual workers might have been essential workers such as in aged‐care or childcare, reportedly poorly paid in Australia., with hours reduced though lockdowns. Analysis demonstrated that some were already struggling financially and, in some cases, recalculated their spending patterns to focus on the immediate needs of food and household utility bills. Other evidence of already struggling financially was that some people were no longer taking medications or attending medical appointments. Loss of employment due to lockdowns led to reliance on government welfare services, borrowing money or using savings. Levels of distress and uncertainty were evident. The response from the person receiving a Disability Pension suggests that lockdowns had less impact on them, though it is likely they felt the impact of rising costs such as rent and food costs. There is evidence from other areas of the survey that respondents were already struggling financially. Wave 5 collected data at the beginning of COVID‐19 lockdowns, demonstrating some respondents were already struggling with not being able to pay utility bills on time, while a larger proportion went without food, or could not pay rent or mortgages. Some responses demonstrated the stress of uncertainty due to lockdowns. Use of terms such as ‘broke,’ ‘survived,’ ‘becoming unemployed,’ ‘no income’; ‘couldn't pay rent’ are examples. Additionally, Wave 5 responses (on properties see the Methods section) provided evidence of people going without meals, being unable to heat the house and relying on welfare organisations for assistance.

**Table 3:** Qualitative responses by income.

During COVID did you have any financial problems?	
Weekly income 1: $0–749 2: 750–1499 3.$1500 or +	Responses
1	Disability pension is very helpful and I would not have survived without it
1	Electricity and water bills. The bonuses and grant for water were most helpful
1	Having to pay to see a Medical Physician to diagnose the herniated slipped discs L4‐S1. Then accessing prescribed care e.g. physiotherapist consults. Thankfully I've already seen a separate Physio for +10 yrs to treat injuries caused by seizures, so they agreed to see me in person. But paying for the MRI, CT guided Epidural Cortisone injections, Radiologist and the Medical Physician during lockdown came with an increased out of pocket cost during lockdown. I had to use savings and ask family if I could gradually pay them back. The COVID benefit lump sum payment helped this.
1	Husband has to have an op at private hospital as public won't take him
1	I am financially broke and regularly have problems with paying bills on time, with buying groceries
1	I became unemployed and couldn't afford the rent or doctor's appointments or medications.
1	I was going through separation. My ex‐husband was taking my income and not allowing me to have it
1	I work part time as a yoga instructor. I couldn't teach classes and earn money because of the restrictions
1	I'm casual. I had no work
1	Just paying for meds was hard. If I could afford it later, I might see the doctor.
1	Less pension!
1	Live below the poverty line—one of the reasons I do not take medications anymore
1	Living with Mum who provided support
1	partner not working due to no work available so financial pressure as only my wage
2	I had no work, then a little bit of work, then had very little money trying to pay my household bills and living expenses.
2	Change of jobs
2	Find it hard to make bills, dental treatments and paying for my medications
2	Husband gave up his customer facing job. We were on one wage and our daughter's Austudy assistance for a few months. I was lucky to have some savings and hubby retrained in between lockdowns.
2	Necessary Medication is expensive
2	Lost employment early covid (4mnths) but gained employment quickly after
2	Once we declared we are partners our disability pension went from $1100 each to approx. $400 but decreases with our wages, leave pay. Thus, only average $90–$180 DSP per fortnight; sometimes without a 2nd wage as partner on unpaid leave or not receiving any parenting payments for over 2 months. With a baby and such decreased wage/income/financial DSP support, it's been frustratingly hard particularly for partner. Her stress stresses me
2	Ongoing mortgage rate increases not aligning with wages
No income recorded	Our entire business had to close so obviously we had to rely solely on Government support
2	Prefer not to say

## 4 Discussion

The results of our study support that GPs can better appreciate the health and lives of PWE by understanding the effects of different levels of income. We showed well‐off PWE were mostly seizure‐free and using fewer ASMs. During COVID‐19 those with the lowest income had most problems in accessing healthcare. Both QoL and Social Support were significantly less for PWE with a low income. This group had three times worse effect of epilepsy on life compared to those well‐off. Our study also showed that 46.2 percent of PWE received Centrelink such as unemployment benefits, disability or aged pensions, with the largest group receiving a Disability pension. Those on Centrelink payments had significantly poorer QoL and more negative effects of epilepsy on the rest of life, but showed no differences in Social Support, contrary to low income PWE. Centrelink payments are consistently well below the ‘poverty line,’ established in 1973 by the Henderson Inquiry [24]. This Inquiry established the income required to meet the basic needs of a family of two adults and two children.

Both the Australian and state governments provided assistance to people on low incomes to manage during the extensive lockdowns, for example New South Wales Relief Grants and Disaster Payments and the Australian Jobseeker program rental assistance. Those PWE on low incomes unable to work during lockdowns found little improvement despite such assistance. This relates to such relief programs only temporarily providing financial assistance to continue living below the poverty line.

One of the major findings of this study was that epilepsy continued to have negative effects on key life aspects, such as on relationships, health, self‐esteem and standard of living for all PWE. These were much worse for PWE who had a low income. Andersson et al. [17] argue that low education and income, used as indicators of low socio‐economic status, have been found to be associated with epilepsy. Conversely those PWE with a higher socio‐economic status, for example, have been found to have greater access to a neurologist.

It is important for GPs to know the effect of income disparities on health. Ziegelstein [25] argues when much of a GP's time is taken up with administration, it is important for them to get to know the patient as a person. In this context knowing the effect of low income in PWEs is very important, for treatment and referral.

Studies across high income countries demonstrate that the results of this study in an Australian survey are not unique. Sadr et al. [26] found that for low SES groups PWE's QoL was lower. Reflecting Marmot's remarks this study demonstrated low income was associated with poorer QoL. It was also associated with financial distress during COVID‐19. Alarmingly, Cihan et al. [27] reported the incidence of SUDEP (sudden death due to epilepsy) was higher in low SES PWE,

Similar findings on the role of employment and incomes for PWE are found in other countries, while Das et al. [28] found that Indian PWE on low incomes discontinued treatment due to its cost. Li et al. [29] found in Sweden, low occupation and low income were associated with greater risk of hospitalisation for epilepsy.

According to Groover et al. [30] there has been scant research on socioeconomic status on PWE and their caregivers. The current study showed the significance of income across a range of variables, particularly that epilepsy had a much larger negative effect on a range of life factors when income was low. This was compared to the well‐off and even those with a moderate income.

Marmot's work on the social gradient has importance here. Inequality has been increasing as shown by Flavel et al. [31] in terms of income levels demonstrating that a social gradient operates in Australia and the impact of such inequality has consequences for health, in this case of PWE.

Sharobeam et al [32] report that during the past 30 years there has been a shift in Australia related to GPs’ attitudes regarding epilepsy, however there are certain shortfalls in knowledge. The GPs surveyed were aware of classifications of epilepsy; they rarely initiated anti‐seizure medicines or diagnostic tests but did alter regimens without referring back to the neurologist. GPs were aware of the need for psychological care and maintained that PWE receiving good care through better control of seizures were able to work. However, there is no recognition that low income is a barrier to both access to diagnosis, treatments and maintaining control. Our research suggests that it is important that those diagnosing and prescribing medications be aware that some PWE may not continue their treatment or keep appointments due to costs.

There are models suggesting that better understanding of the barriers created by low income may improve GPs knowledge of the needs of PWE For example in Scotland the Deep End Project was established in 2016. It is based on a collaboration of academic and practice GPs servicing the most socio‐economically deprived groups. It was formed in order for low‐income populations to increase recruitment and retention of GPs, undertaken so thar the participating GPs better understand the needs of this group [33]. This model could be applied to consider access issues to epilepsy care for PWE.

The results of this Australian survey and additional evidence provided internationally of the barriers created by low income to accessing diagnosis and continuing care strongly suggest that income should be taken into account when GPs are considering the best treatment for a PWE. This is even more important for people from rural and remote areas, where the cost of travel to a specialist centre might be out of reach.

## 5 Limitations

One limitation was the sample size which meant the results would have had a greater impact if there were larger numbers. An aspect of this was the small numbers providing open‐ended written responses. Further, with regard to open‐ended responses, these provided evidence of the stress PWE on low incomes and job‐loss faced. However, lengthier responses might have produced even more evidence regarding the inability to manage their well‐being and access healthcare, plus any coping mechanisms.

## 6 Conclusion

The AIHW data demonstrate that PWE attend GPs and receive prescriptions. However, there is little evidence of how epilepsy is managed in General Practice. At the same time, inequality, in this case, low income, demonstrates that some Australian PWE are at risk of abandoning epilepsy treatment altogether if their needs are not well understood.

While recognising the serious challenges faced by PWE in low‐income countries as pointed out by the ILAE [4] this research contributes to the social inequalities existing in high‐income countries. It covers the period where PWE coped with the social and income constraints introduced to deal with the COVID‐19 pandemic. Responses from survey show that PWE, already stressed due to low incomes, were unable to attend medical appointments, afford ASMs and had to economise to buy food and pay bills.

The solution provided by the ILAE is to educate GPs to better diagnose, care for PWE and make referrals to specialists. This goes some way to providing more affordable care for PWE on low incomes. Receiving good care over extended periods may help some PWE to better paid employment. However, what remains to be addressed are the significant barriers for PWE who cannot afford to access even GP care due to low income. Awareness of these barriers might be part of a medical curricula.

## Conflicts of Interest

The authors declare no conflicts of interest.

## Data Availability

Research data are not shared.
